# The Administration of Cortisol Induces Female-to-Male Sex Change in the Protogynous Orange-Spotted Grouper, *Epinephelus coioides*

**DOI:** 10.3389/fendo.2020.00012

**Published:** 2020-01-31

**Authors:** Jiaxing Chen, Cheng Peng, Zeshu Yu, Ling Xiao, Qi Yu, Shuisheng Li, Haifa Zhang, Haoran Lin, Yong Zhang

**Affiliations:** ^1^State Key Laboratory of Biocontrol, Guangdong Provincial Key Laboratory for Aquatic Economic Animals and Southern Marine Science and Engineering Guangdong Laboratory (Zhuhai), School of Life Sciences, Sun Yat-sen University, Guangzhou, China; ^2^Laboratory for Marine Fisheries Science and Food Production Processes, Qingdao National Laboratory for Marine Science and Technology, Qingdao, China; ^3^Guangdong Key Laboratory of Animal Conservation and Resource Utilization, Guangdong Public Laboratory of Wild Animal Conservation and Utilization, Guangdong Institute of Applied Biological Resources, Guangzhou, China; ^4^Southern Marine Science and Engineering Guangdong Laboratory (Zhanjiang), Fisheries College, Guangdong Ocean University, Zhanjiang, China; ^5^Marine Fisheries Development Center of Guangdong Province, Huizhou, China

**Keywords:** cortisol, grouper, masculinization, protogynous, sex change

## Abstract

In this study, we injected cortisol into the protogynous orange-spotted grouper (*Epinephelus coioides*) to investigate the role of this hormone in sex change. Following injection, we evaluated gonadal changes, serum levels of steroid hormones, and sex-related gene expression during the processes of cortisol-induced sex change and cortisol withdrawal in the orange-spotted grouper. Cortisol treatment caused the degeneration of oocytes and induced sex change in a dose-dependent manner. Over the long-term, we observed a significant increase in serum 11-ketotestosterone (11-KT) levels in all cortisol-treated groups, although levels of 17β-estradiol did not change significantly. Consistent with the elevation of serum 11-KT levels, the expression of genes related to testicular development was also significantly up-regulated in the cortisol-treated groups. Based on our results, we propose that cortisol may trigger masculinization by inducing the synthesis of 11-KT and by directly activating the expression of sex-related genes. Furthermore, we found that cortisol-induced sex change was not permanent and could be reversed after the withdrawal of cortisol treatment.

## Introduction

Teleost fish exhibit remarkably diverse and plastic patterns of sexual determination and differentiation. In most gonochoristic fishes, the determination of sex is genetic, while in other cases, sex is determined by environmental factors such as social conditions, temperature, pH, and hypoxia (1). Furthermore, some teleosts exhibit natural sequential hermaphroditism; in other words, an individual changes from one sex to the other as an adaptive part of their life cycle (2). However, the regulatory mechanisms associated with this adaptive response have yet to be elucidated.

In recent years, cortisol, the major glucocorticoid in fish, has been proposed as a mediator linking external environmental stimuli with processes responsible for internal masculinization (3). In several gonochoristic species, including pejerrey (*Odontesthes bonariensis*) (4), Japanese flounder (*Paralichthys olivaceus*) (5), and medaka (*Oryzias latipes*) (6), the elevation of serum cortisol levels has been shown to be correlated with high temperature-induced masculinization.

In protogynous hermaphroditic fish, cortisol is also involved in the initiation of sex change. In the bluehead wrasse (*Thalassoma bifasciatum*), serum cortisol level is associated with social hierarchical status; elevated levels of serum cortisol were observed in subordinate females and were thought to prevent protogynous sex change (7). However, a significant increase in cortisol levels was observed during the early stages of sex change in the protogynous blue-banded goby (*Lythyrpnus dalli*) (8). Interestingly, in our previous study, a spike in serum cortisol levels was observed during the initiation of sex change in the orange-spotted grouper (unpublished data). Collectively, these data indicate that cortisol may play a critical role in triggering protogynous sex change.

In teleosts, two key enzymes, 11β-hydroxylase (Cyp11b) and 11β-hydroxysteroid dehydrogenase (Hsd11b), have been proposed to play critical roles in the cross-talk between glucocorticoid and androgen pathways by regulating the synthesis of androgens and the metabolism of glucocorticoids (9). In the Japanese eel (*Anguilla japonica*), cortisol was shown to induce spermatogonial mitosis in testicular fragments by increasing the production of 11-ketotestosterone (11-KT) (10). During the high-temperature induction of masculinization in the pejerrey (*Odontesthes bonariensis*), cortisol was suggested to enhance 11-KT synthesis by modulating the expression of *hsd11b2* and therefore driving the subsequent morphogenesis of the testes (11). In some protogynous fish, a precipitous drop in serum 17β-estradiol (E2) levels is regarded as the initiation of gonadal sex change (12, 13). Other studies have shown that an increase in the levels of circulating cortisol could directly inhibit the production of E2 by binding to glucocorticoid receptors (GRs) and subsequently interacting with the glucocorticoid responsive element (GRE) within the promoter region of *cyp19a1a* to suppress gene transcription (5, 12). It is also possible that cortisol may mediate protogynous sex change by upregulating the expression of male-related genes such as *amh*. In a previous study, a significant increase in *amh* expression was observed during temperature-induced masculinization in both Japanese flounder (14) and Nile tilapia (*Oreochromis niloticus*) (15).

The orange-spotted grouper is a typical protogynous hermaphroditic fish and is an important mariculture species that is widely cultured in southern Asia (16). Most orange-spotted grouper become mature at 4 to 5 years of age as a female, and some females can then change sex to males later in their life (17). In orange-spotted grouper, sex is controlled primarily by social factors and can also be induced under experimental conditions by isolating mature females (18). Previous studies have already described the gonadal changes, serum sex steroid hormone levels, and sex-related gene expression patterns in this species during sex change (16, 17). Hence, the orange-spotted grouper is an excellent model for investigating the physiological mechanisms related to sex change. In the present study, we aimed to investigate the role of cortisol in protogynous sex change and identify the physiological mechanisms that regulate sex change by administering cortisol to groups of matured orange-spotted grouper.

## Materials and Methods

### Animals

Four-year-old mature male and female orange-spotted groupers were obtained from the Marine Fisheries Development Center of Guangdong Province (Huizhou, China). Before the experiment, all fish were cannulated through the genital pore to collect gonadal tissue and examined by histology to determine gonadal stage. Fish were individually tagged with a biochip transponder under the dorsal skin for identification. The body weight (BW) and standard length (SL) of each fish were also recorded. All animal handling and experimentation were conducted in accordance with the guidelines and approval of the Animal Research and Ethics Committees of Sun Yat-sen University.

### Experimental Design

#### Experiment 1

Sixteen females (3.85 ± 0.14 kg in BW and 49.96 ± 0.71 cm in SL) with primary oocytes were selected and randomly divided into four groups: a control group, a low-dose cortisol group, a medium-dose cortisol group, and a high-dose cortisol group. Four males (4.14 ± 0.16 kg in BW and 52.45 ± 0.84 cm in SL), as identified by their ability to spermiate, were selected and separately placed into each group to suppress the socially induced sex change among females. The four groups were kept separately in concrete tanks fed by flow-through seawater under a natural photoperiod and fed once daily with frozen fish. Cortisol (Sigma-Aldrich, St. Louis, MO, USA) was dissolved in dimethyl sulfoxide and diluted 10 times in 0.9% saline. The fish were anesthetized using MS-222 (Sigma-Aldrich, St. Louis, MO, USA) and then intraperitoneally injected with cortisol at a dose of 2 mg/kg body weight (low-dose group), 10 mg/kg body weight (medium-dose group), 50 mg/kg body weight (high-dose group), and 10% dimethyl sulfoxide (control group) every 5 days from 19th August 2018 to 17th November 2018. To determine the short- and long-term effects of cortisol injection on sex change, we acquired blood samples from each fish at 12, 24, 48, and 96 h after treatment (hat) and 7, 15, 30, and 60 days after treatment (dat) for serum steroid assays. Gonadal tissues were collected from the females in each group by cannulation at several sampling time points (24 hat and 96 hat for RNA isolation; 7, 15, 30, and 60 dat for RNA isolation and histological analysis).

#### Experiment 2

To investigate the influence of cortisol withdrawal on sex change, another 16 females (3.51 ± 0.07 kg in BW and 47.56 ± 0.48 cm in SL), along with four males (4.33 ± 0.07 kg in BW and 52.05 ± 0.68 cm in SL), were selected and randomly divided into four groups. In two groups, we stopped high-dose cortisol injection (50 mg/kg body weight) after 30 and 60 days, respectively. A third group received high-dose cortisol injection until 90 dat as a positive control, while a fourth group received a vehicle injection (10% dimethyl sulfoxide) as a negative control. Blood samples and gonadal tissues were acquired from the fish at 60 and 90 dat (30 days after cortisol-injection withdrawal in the two cortisol-withdrawal groups).

### Body Indices

Gonadsomatic index (GSI) was determined for all sacrificed fish according to the following formula: GSI = 100 (gonadal weight/total weight).

### Gonadal Histology

Pieces of gonadal tissues were fixed in Bouin's fluid for 24 h at room temperature and then transferred to 70% ethanol prior to dehydration and paraffin embedding. Gonadal tissues were then serially sectioned at a thickness of 7 μm and stained with hematoxylin and eosin for subsequent analysis. Gonadal stage and sex change status were then determined under light microscopy (LEICA DM 1000 LED, Wetzlar, Germany).

### Serum Steroid Hormone Assays

Approximately 1 ml of blood was collected from the caudal vein of each fish using a 5 ml non-heparinized syringe and kept in a 1.5 ml microcentrifuge tube until being centrifuged ~3 h later. Serum was separated by centrifugation at 6,000 g for 10 min and then stored at −20°C until further use. The levels of serum cortisol, 11-KT, and E2 were subsequently measured using Enzyme Immunoassay Assay kits (Cayman Chemical Co, Ann Arbor, MI, USA) according to the manufacturer's instructions.

### RNA Isolation, Reverse Transcription, and Quantitative Real-Time PCR

Total RNA was extracted from the tissue samples using TRIzol reagent (Invitrogen, Carlsbad, CA, USA) and reverse transcribed into cDNA by ReverTra Ace qPCR RT Kit (TOYOBO, Osaka, Japan) in accordance with the manufacturer's instructions. Real-time PCR was performed on a Roche Light Cycler 480 real-time PCR System using SYBR Green Real-Time PCR Master Mix (Roche, Germany) according to the manufacturer's protocol. The real-time PCR conditions were as follows: denaturation at 95°C for 5 min, followed by 40 cycles at 95°C for 15 s, 56°C for 20 s, and 72°C for 20 s. The mRNA levels of *gr1* (glucocorticoid receptor 1), *gr2* (glucocorticoid receptor 2), *dmrt1* (doublesex and mab-3-related transcription factor 1), *sox9* (sex-determining region y-box 9), *amh* (anti-Müllerian hormone), *hsd11b2* (11β-hydroxysteroid dehydrogenase type 2), *cyp11b* (11β-hydroxylase), and *cyp19a1a* (aromatase P450) were then analyzed, with β*-actin* serving as an internal control. After amplification, fluorescent data were converted to threshold cycle values (Ct). The relative abundance of mRNA transcripts was then evaluated using the formula R = 2^−ΔΔCt^, as described previously (19). The sequences encoding for the genes investigated in this study were obtained from transcriptomic data (unpublished data). Table 1 lists the primers used in this study.

**Table 1:** Nucleotide sequences of the primers used in this study.

**Primers**	**Primer sequence (from 5′ to 3′)**
**Primers for real-time PCR**	
*gr1*-QF	TACCGAAAGATGGCCTGAAAA
*gr1*-QR	TCCTGCATGGAGTCCAATAGC
*gr2*-QF	GCAGGCTAACCACAGAGGGC
*gr2*-QR	GCGTGAAAGGGACTGGAATG
*dmrt1*-QF	GCTGGAGTAGACTGCTTGTTT
*dmrt1*-QR	CGACTGTGCGTCAGTATGAGC
*sox9*-QF	GCAATGCAGGCTCAGAATAG
*sox9*-QR	GGTATCAAGGCAGTACCCAG
*amh*-QF	TGTTGGGAGCGACGGTGAACT
*amh*-QR	TGCAGCGACTGACTCGTGAAA
*hsd11b2*-QF	TCTGGGCTTTGAGGTGTTCG
*hsd11b2*-QR	TGGATCTGCTGTGGTTGGGT
*cyp11b*-QF	TACAGGTGTTGGAAAGAAGG
*cyp11b*-QR	CTCTCCAGAACTTCACCAAG
*cyp19a1a*-QF	ACAGTGGAGGTCAGTGTCTC
*cyp19a1a*-QR	GACAGGTACATCCAGGAAGA
β-*actin-QF*	ACCATCGGCAATGAGAGGTT
β-*actin-QR*	ACATCTGCTGGAAGGTGGAC
**Primers for cloning**	
*gr1*-F	ATGGATCAGGGTGGACTGAAG
*gr1*-R	TTTCTGATGAAACAGCAGAGGC
*gr2*-F	ATGGATAACAGTGGAGTGAAG
*gr2*-R	TCTCTGGTGGAAGAGGAGAG
*amh* promoter-F	TGGCCTAACTGGCCGGTACCTCCAAATGCTGCTTCA CTCA
*amh* promoter-R	TCTTGATATCCTCGAGGCTTCACTGTCTGTACGTCT
*cyp19a1a* promoter-F	CGGGGTACCGAGGAGTTGATAAATTCTGTTCCGAC
*cyp19a1a* promoter-R	CCGCTCGAGCACAAGCAGAGATGAGATCCATAAGAA

### Terminal Deoxynucleotidyl Transferase dUTP Nick End Labeling (TUNEL) Assay

Apoptosis during cortisol-induced sex change was detected using a TUNEL Apoptosis Detection Kit (Phygene, Fuzhou, China) in accordance with the manufacturer's instructions. Samples were then analyzed under a light microscope (Nikon IQ50, Tokyo, Japan).

### Cell Culture, Transient Transfections, and Dual-Luciferase Assay

Based on genomic and transcriptomic data (unpublished data) previously obtained for the orange-spotted grouper, we amplified the complete open reading frame (ORF) of *gr1* and *gr2* using Phanta Max Super-Fidelity DNA Polymerase (Vazyme Biotech, China) and then inserted the ORF into the pcDNA4.0 vector (Invitrogen). Human embryonic kidney (HEK) 293 cells were then cultured in DMEM (Hyclone, USA) supplemented with 10% fetal bovine serum (FBS) (Hyclone, USA) at 37°C in a humidified atmosphere containing 5% CO_2_. To confirm the expression of *gr1* and *gr2* in HEK293 cells, the pcDNA4.0-gr1 and pcDNA4.0-gr2 plasmids were transfected into HEK293 cells using Lipofectamine 3000 reagent (Invitrogen), respectively. At 24 h after transfection, the cells were lysed with RIPA lysis buffer (Beyotime Institute of Biotechnology, China) containing 1% protease inhibitor (Sigma-Aldrich, St. Louis, MO, USA), and total proteins were extracted for Western blotting using an anti-his tag antibody (Proteintech, USA).

To analyze ligand specificity and the downstream signaling pathways of *gr1* and *gr2*, HEK293 cells were seeded into 48-well plates and cultured 24 h prior to transfection. Then, 200 ng/well pcDNA4.0-*gr1*/pcDNA4.0-*gr2*, 100 ng/well pGRE-Luc (Beyotime Institute of Biotechnology, China) and 5 ng/well pRL-TK (Promega, WI, USA) were co-transfected. Approximately 12 h after the transfection, the medium was replaced with fresh medium (supplemented with 10% FBS). We then treated the transfected cells with cortisol (Sigma-Aldrich, St. Louis, MO, USA). Luciferase activity was measured after 12 h of hormone treatment using the Dual-Luciferase Reporter Assay System (Promega) in accordance with the manufacturer's instructions.

To confirm whether cortisol was able to control the relative expression of *cyp19a1a* and *amh* by binding to GREs within the promoter regions, we amplified a 2,500 bp sequence upstream from the translational start site of *cyp19a1a* (GenBank Accession Number: JF420889) and *amh* (GenBank Accession Number: MG017511) and inserted these fragments into the pGL4.1 vector (Invitrogen) using *KpnI* and *XhoI* restriction sites. HEK293 cells were then seeded into 48-well plates and cultured for 12 h. Cells were then co-transfected with 200 ng/well of pcDNA4.0/pcDNA4.0-*gr1*/pcDNA4.0-*gr2*, 100 ng/well pGL4.1/pGL4.1-*cyp19a1a*/pGL4.1-*amh*, and 5 ng/well pRL-TK. Approximately 12 h after transfection, cells were treated with cortisol (1,000 ng/ml). Luciferase activity was measured 12 h later. All experiments were repeated independently three times. Table 1 shows the primers used for cloning.

### Statistical Analyses

Data are expressed as the mean ± standard error of the mean (SEM). Differences between groups were evaluated by means of one-way analysis of variance (ANOVA), followed by the Tukey test with statistical significance set at *P* < 0.05. All statistical tests were performed using SPSS 18.0 (SPSS, Chicago, IL, USA).

## Results

### Gonadal Histology During Cortisol-Induced Female-to-Male Sex Change

**Figure 1:**
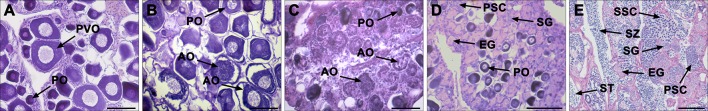
Gonad histology during cortisol-induced sex change from female to male in the orange-spotted grouper. **(A)** Gonad histology of a female with oocytes. **(B)** Gonad histology of a female at the early stage of degeneration with atretic oocytes. **(C)** Gonad histology of a female at the late stage of degeneration, with oocytes undergoing further degeneration. **(D)** Gonad histologyes of an intersex-transitional phase individual, with the presence of spermatogenic germ cells at various developmental stages and the oocytes in primary growth. **(E)** Gonad histology of a sex-changed male with active spermatogenesis. AO, atretic oocyte; EG, early germ cell; PO, primary oocyte; PSC, primary spermatocytes; PVO, previtellogenic oocyte; SG, spermatogonia; SSC, second spermatocytes; ST, spermatid; SZ, spermatozoa. Scale bars, 50 μm.

Gonadal reprogramming of cortisol-induced female-to-male sex change can be divided into four phases: a female phase, a degenerative phase, an intersex-transitional phase, and a male phase. In brief, the female phase was characterized by the presence of primary oocytes and previtellogenic (cortical alveolar) oocytes in the ovary (Figure 1A). During the degenerative phase, the ovary underwent degeneration and contained numerous atretic oocytes (Figures 1B,C). The intersex-transitional phase, in which female and male germ cells coexisted in the gonad, was characterized by the degeneration of oocytes and a simultaneous proliferation of spermatogonia in spermatogenic cysts (Figure 1D). During the male phase, spermatogenic germ cells were evident in the gonad at various stages of development (Figure 1E). The gonadal stages of fish in the different experimental groups are shown in Table 2.

**Table 2:** Gonadal stage of fish during the cortisol-injection experiment.

**Groups**	***N***	**Time points of sampling**																			
		**Day 0**				**Day 7**				**Day 15**				**Day 30**				**Day 60**			
		**Gonadal stage**																			
		**F**	**D**	**I**	**M**	**F**	**D**	**I**	**M**	**F**	**D**	**I**	**M**	**F**	**D**	**I**	**M**	**F**	**D**	**I**	**M**
Control	4	4				4				4				4				4			
Low-dose	4	4					4			4				4				4			
Medium-dose	4	4					4				4				2	2			2	2	
High-dose	4	4					4				4					4				2	2

*F, female phase; D, degenerative phase; I, intersex-transitional phase; M, male phase*.

During the experimental period, the gonads of fish in the control group retained their ovarian status. However, in the groups receiving cortisol injection, the gonads showed structural changes. At 7 dat, all of the cortisol-injection groups were undergoing the degenerative phase and contained many atretic oocytes. At 15 dat, the ovaries had undergone further degeneration, and almost all of the atretic oocytes had been absorbed in the high-dose cortisol and medium-dose cortisol groups. At 30 dat, two fish in the medium-dose cortisol group and all the fish in the high-dose cortisol group had entered the intersex-transitional phase, suggesting that sex change was underway. By 60 dat, two fish in the high-dose cortisol group were found to possess mature testes, indicating that sex change had been completed, and the gonads of the remaining two fish still remained in the intersex-transitional phase. Although atretic oocytes were observed in the low-dose cortisol group, no sex change occurred, and oocytes remained in the primary growth stage throughout the experimental period.

**Figure 2:**
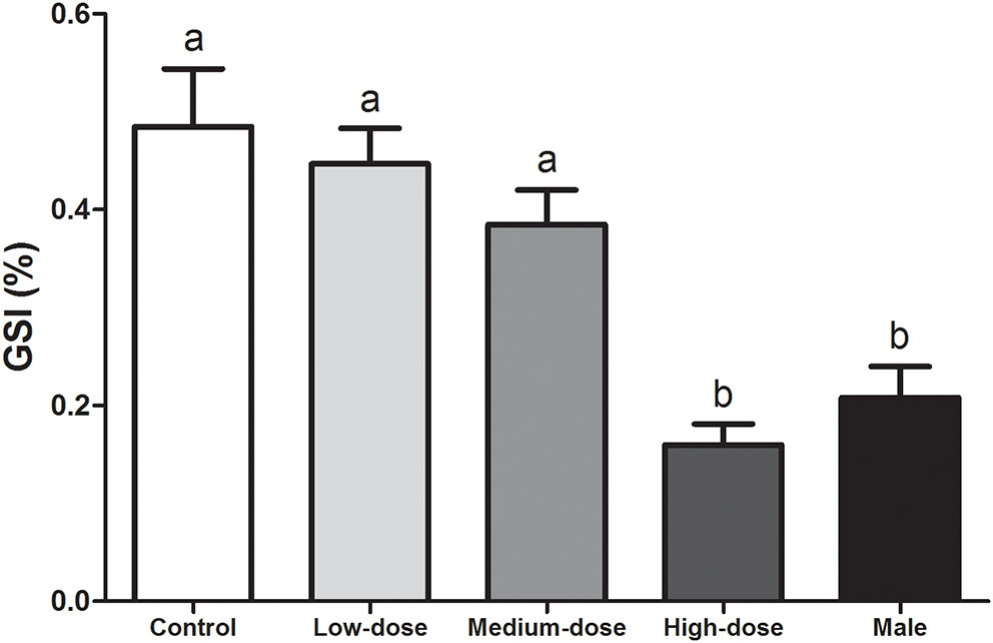
Effects of cortisol administration on the gonadosomatic index (GSI) of the orange-spotted grouper. GSI of the fish in the control group, cortisol-treated groups (low-dose cortisol group, medium-dose cortisol group, and high-dose cortisol group), and males at completion of the experiment (60 days after treatment). Data are presented as the mean ± SEM of four fish. Different letters indicate significant differences (*P* < 0.05).

By the end of the experiment, the GSI of the high-dose group (0.16 ± 0.02%) was significantly lower than that of the other groups (control group, 0.48 ± 0.06%; low-dose group, 0.45 ± 0.04%; and medium-dose group, 0.62 ± 0.04%) and was not significantly different from that of the normal males (0.21 ± 0.03%) (Figure 2).

### Apoptosis During Cortisol-Induced Sex Change

**Figure 3:**
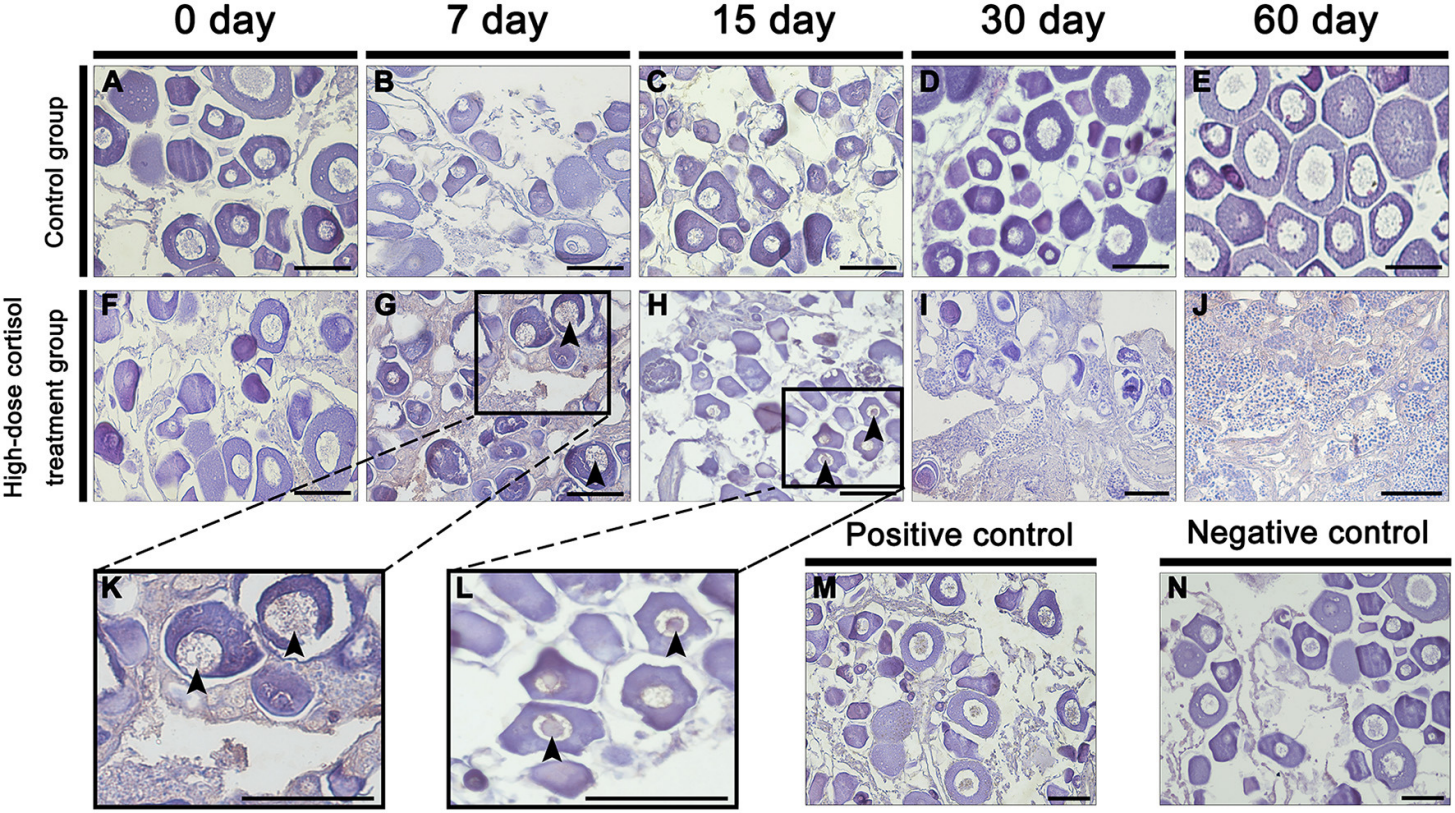
Apoptosis detection during cortisol-induced sex change from female to male in the orange-spotted grouper. **(A–E)** Gonad apoptosis detection of fish in the high-dose cortisol group at different sampling times. **(F–J)** Gonad apoptosis detection of fish in the control group at different sampling times. **(K,L)** Show high magnification views of boxed areas in **(G,H)**. **(M,N)** are positive and negative controls. Black triangular arrow indicates an apoptotic signal. Scale bars, 50 μm.

In the control group, no apoptosis was detected during the experiment (Figures 3A–E). In contrast, gonads in the high-dose cortisol group showed apoptotic signals first at 7 dat in some atretic oocytes (Figures 3F,G,K), and the extent of apoptosis had increased in nearly all the oocytes by 15 dat (Figures 3H,L). However, there was no evidence of apoptosis from 30 to 60 dat (Figures 3I,J), when the gonads entered the intersex-transitional and male phases.

### Serum Steroid Hormone Levels During Cortisol Treatment

**Figure 4:**
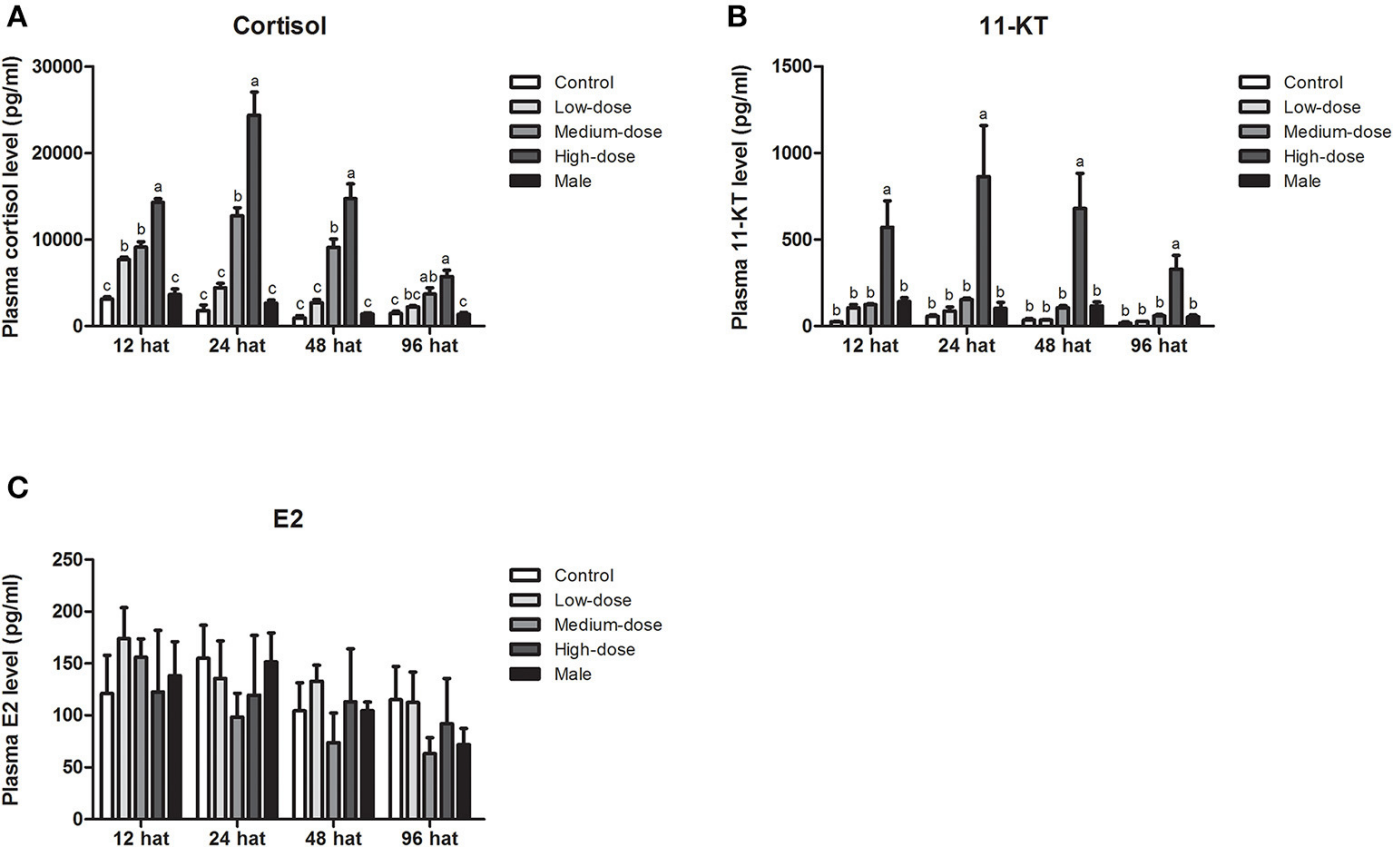
Short-term changes in serum steroid hormone levels after injection of cortisol. Short-term changes of serum **(A)** cortisol levels, **(B)** 11-KT levels, and **(C)** E2 levels in the control group, low-dose cortisol group, medium-dose cortisol group, high-dose cortisol group, and normal males at 12, 24, 48, and 96 h after treatment (hat). Data from four fish samples are expressed as the mean ± SEM. Different letters above the bars indicate statistically significant differences between treatments at the same sampling time (*P* < 0.05).

Next, we analyzed short-term changes in serum steroid hormone levels after cortisol injection. A significant increase in serum cortisol levels was observed in the high-dose and medium-dose cortisol groups at 12, 24, 48, and 96 hat and in the low-dose cortisol group at 12 hat (Figure 4A). Compared with the other groups, serum 11-KT levels were significantly higher in the high-dose cortisol group at 12, 24, 48, and 96 hat (Figure 4B). In contrast, there were no significant differences in terms of E2 levels when compared across the different groups after cortisol treatment (Figure 4C).

During long-term cortisol treatment, we observed an elevation of serum cortisol levels in all of the cortisol-treated groups. Furthermore, cortisol levels in the high-dose and medium-dose cortisol groups were significantly higher than those in the control group at all sampling time points (7, 15, 30, and 60 dat), but in the low-dose cortisol group, serum cortisol levels were only significantly higher than those in the control group at 30 dat (Figure 6A). The changes in serum 11-KT levels were consistent with the changes observed in cortisol levels. At each sampling time, serum 11-KT levels in the high-dose cortisol group were significantly higher than those in the other groups and even higher than the levels of males. In the medium-dose cortisol group, serum 11-KT levels were significantly higher than those in the control group at 15 and 60 dat. No significant difference was observed between the low-dose cortisol group and the control group (Figure 6B). In contrast, serum E2 levels showed no significant difference when compared across different groups at most sampling time points; we only observed a significant reduction of E2 in the high-dose cortisol group at 30 dat and 60 dat (Figure 6C).

### Gene Expression Profiles in the Gonad During Cortisol Treatment

**Figure 5:**
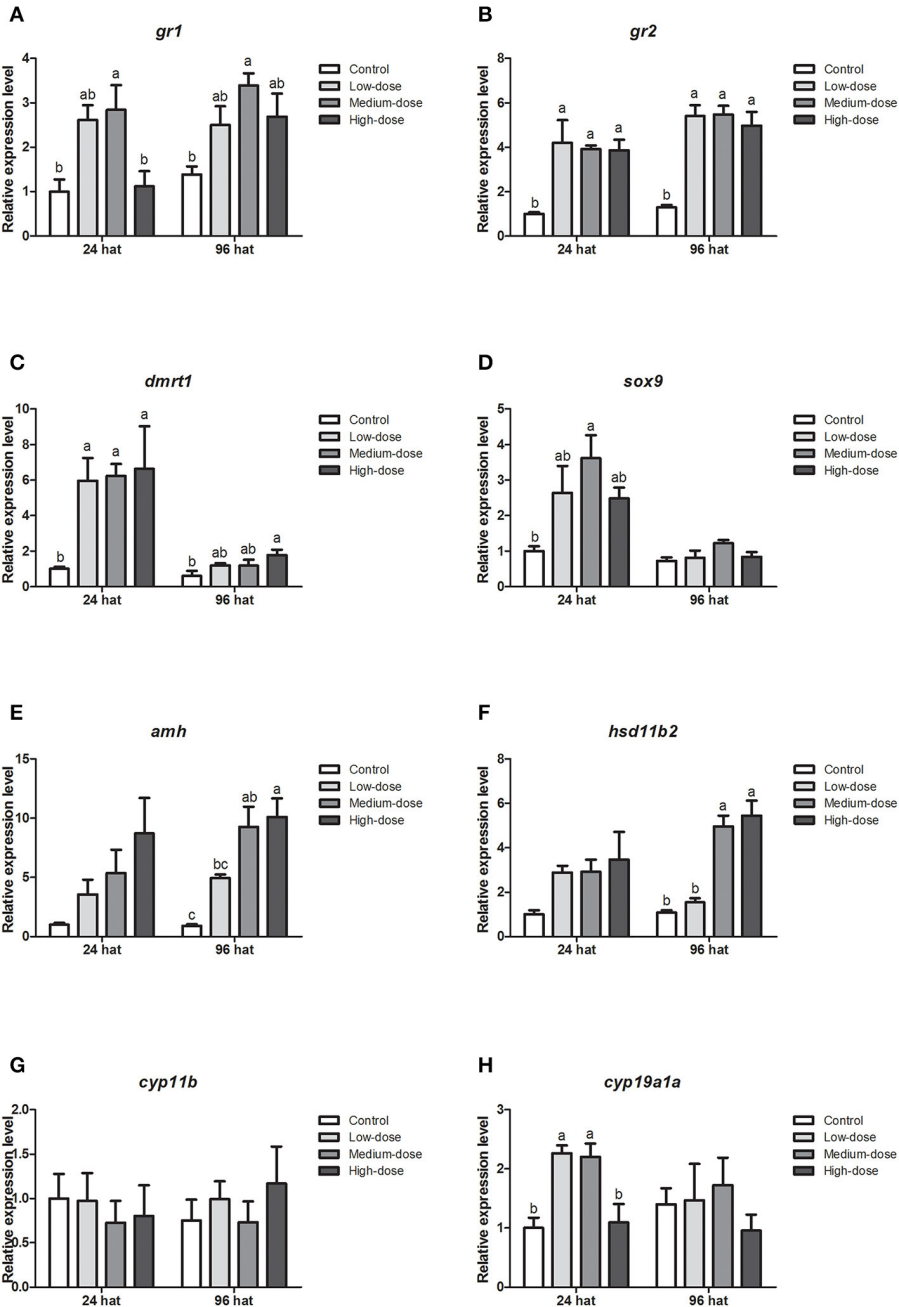
Short-term changes in expression profiles of key genes related to sex change in the gonad after injection of cortisol. **(A,B)** Expression profiles of glucocorticoid receptor genes *gr1* and *gr2* at 24 and 96 h after treatment (hat), respectively. **(C–G)** Expression profiles of male-related genes *dmrt1, sox9, amh, hsd11b2*, and *cyp11b* at 24 and 96 hat, respectively. **(H)** Expression profiles of female-related gene *cyp19a1a* at 24 and 96 hat. β*-actin* was used as the internal control. Data from four fish samples are expressed as the mean ± SEM for three replicates obtained from the samples. Different letters above the bars indicate statistically significant differences between treatments at the same sampling time (*P* < 0.05).

Next, we analyzed the short-term effects of cortisol injection on the gonadal expression of glucocorticoid receptor genes and sex-related genes. The expression of glucocorticoid receptor genes (*gr1, gr2*) and male-related genes (*dmrt1, sox9, amh* and *hsd11b2*) increased rapidly after cortisol injection (Figures 5A–F). However, *cyp11b*, the gene encoding the key enzyme responsible for synthesizing the androgen 11-KT, did not change significantly after the injection of cortisol (Figure 5G). The expression of the female-related gene *cyp19a1a* increased significantly in the low-dose and medium-dose cortisol group at 24 hat but showed no significant difference between the cortisol-treated groups and the control group at 96 hat (Figure 5H).

**Figure 6:**
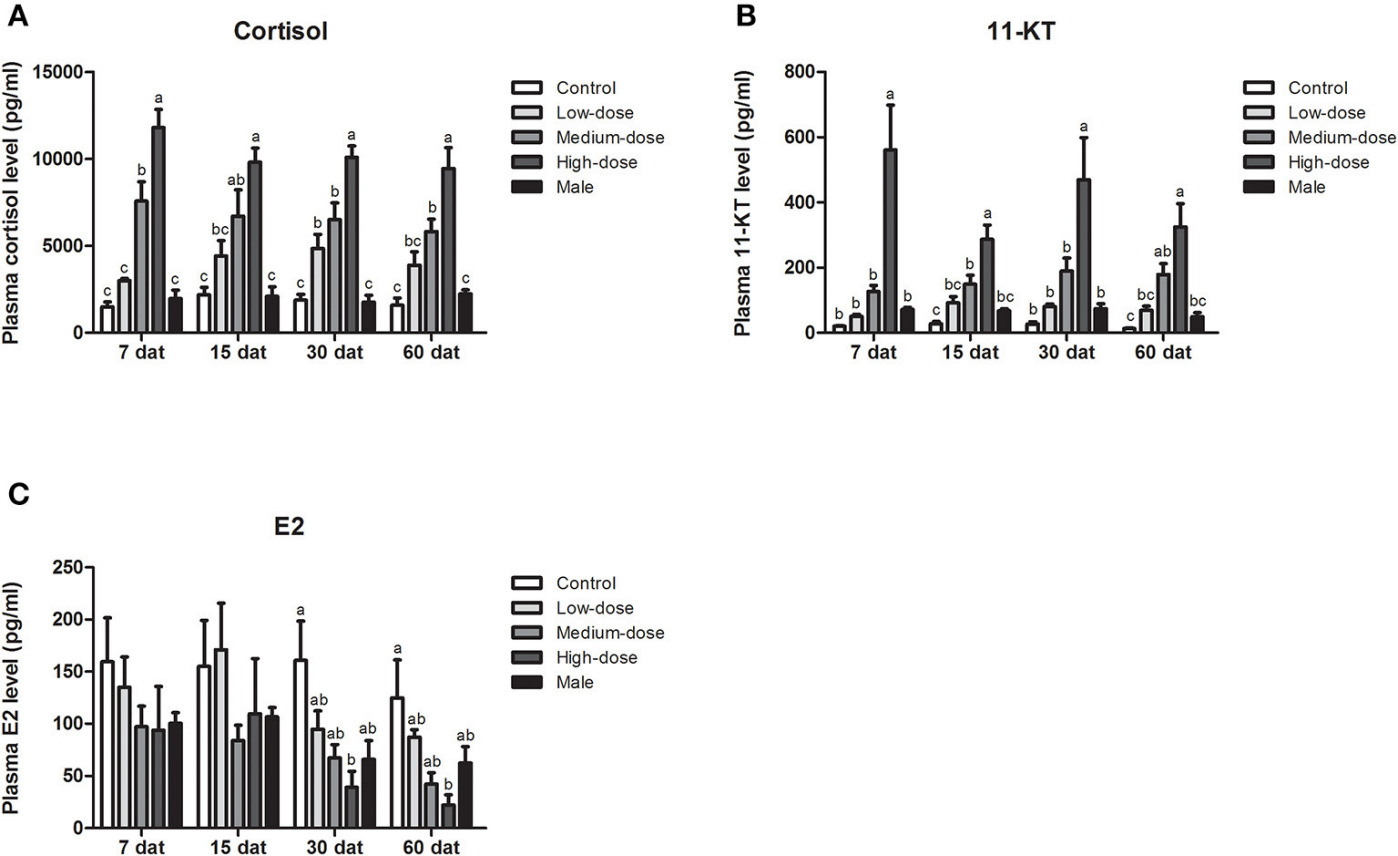
Serum steroid hormone levels during long-term cortisol treatment. Changes in serum **(A)** cortisol levels, **(B)** 11-KT levels, and **(C)** E2 levels in the control group, low-dose cortisol group, medium-dose cortisol group, high-dose cortisol group, and normal males at 7, 15, 30, and 60 days after treatment (dat). Data from four fish samples are expressed as the mean ± SEM. Different letters above the bars indicate statistically significant differences between treatments at the same sampling time (*P* < 0.05).

**Figure 7:**
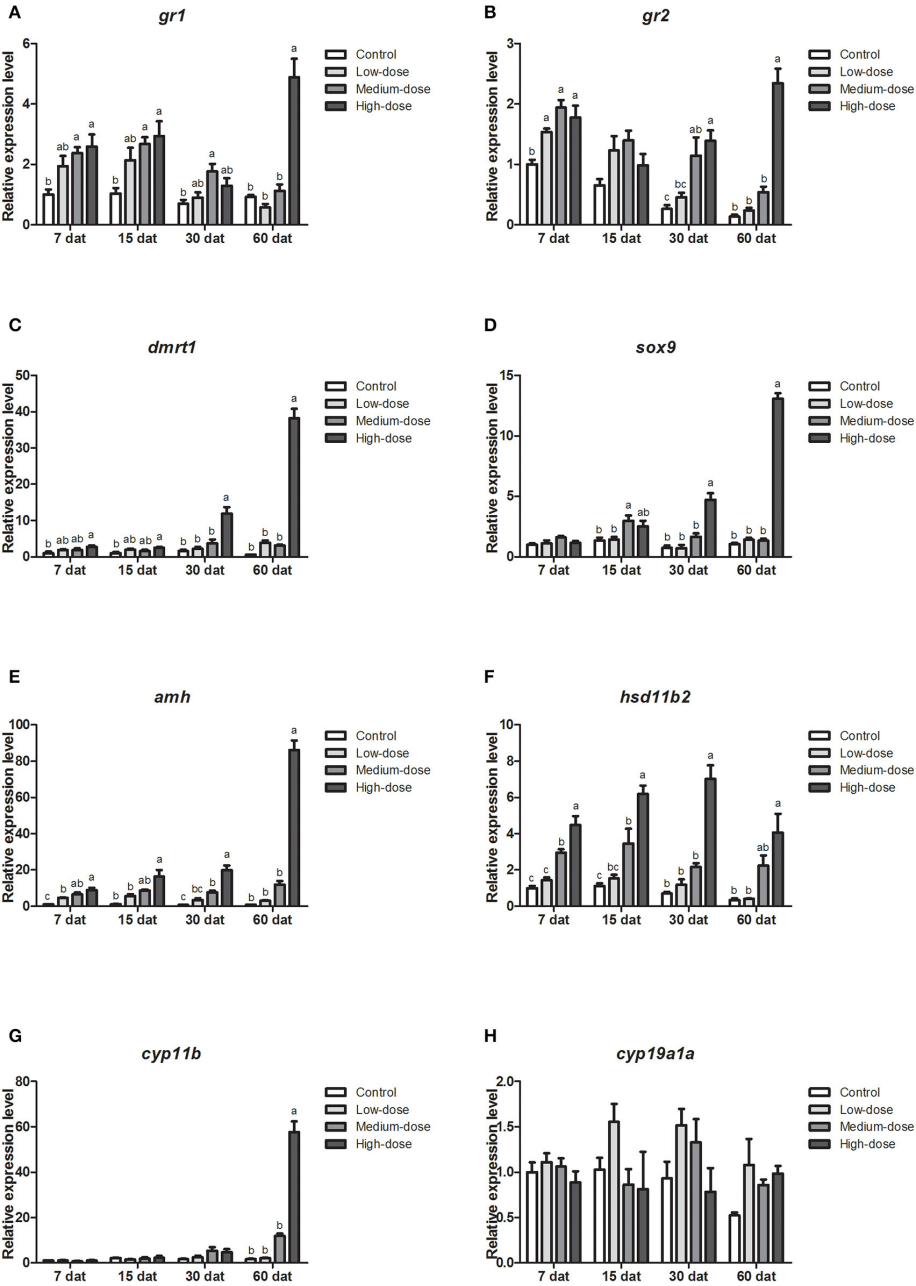
Expression profiles of key genes related to sex change in the gonad during long-term cortisol treatment. **(A,B)** Expression profiles of glucocorticoid receptors genes *gr1* and *gr2* at 7, 15, 30, and 60 days after treatment (dat), respectively. **(C–G)** Expression profiles of male-related genes *dmrt1, sox9, amh, hsd11b2*, and *cyp11b*, at 7, 15, 30, and 60 dat, respectively. **(H)** Expression profiles of female-related gene *cyp19a1a* at 7, 15, 30, and 60 dat. β*-actin* was used as the internal control. Data from four fish samples are expressed as the mean ± SEM for three replicates obtained from the samples. Different letters above the bars indicate statistically significant differences between treatments at the same sampling time (*P* < 0.05).

We also analyzed gene expression profiles during long-term cortisol treatment. Compared with the control group, the expression levels of the glucocorticoid receptor genes (*gr1, gr2*) increased in all of the cortisol-treated groups (Figures 7A,B). The expression of *dmrt1* increased significantly in the high-dose cortisol group from 7 to 60 dat (Figure 7C). Similarly, the expression of *sox9* increased significantly in the high-dose cortisol group from 30 to 60 dat and in the medium-dose group at 15 dat (Figure 7D). We also observed a significant increase in *amh* expression in the high-dose cortisol group from 7 to 60 dat, in the medium-dose group from 7 to 30 dat, and in the low-dose group from 7 to 15 dat (Figure 7E). The expression of *hsd11b2* increased significantly in the high-dose cortisol group from 7 to 60 dat and in the medium-dose group from 7 to 15 dat (Figure 7F). In contrast, the expression of *cyp11b* only increased significantly in the high-dose cortisol group at 60 dat (Figure 7G). It is noteworthy that the expression of the female-related gene *cyp19a1a* did not change significantly during long-term cortisol treatment (Figure 7H).

### Gonadal Histology After the Withdrawal of Cortisol

**Figure 8:**
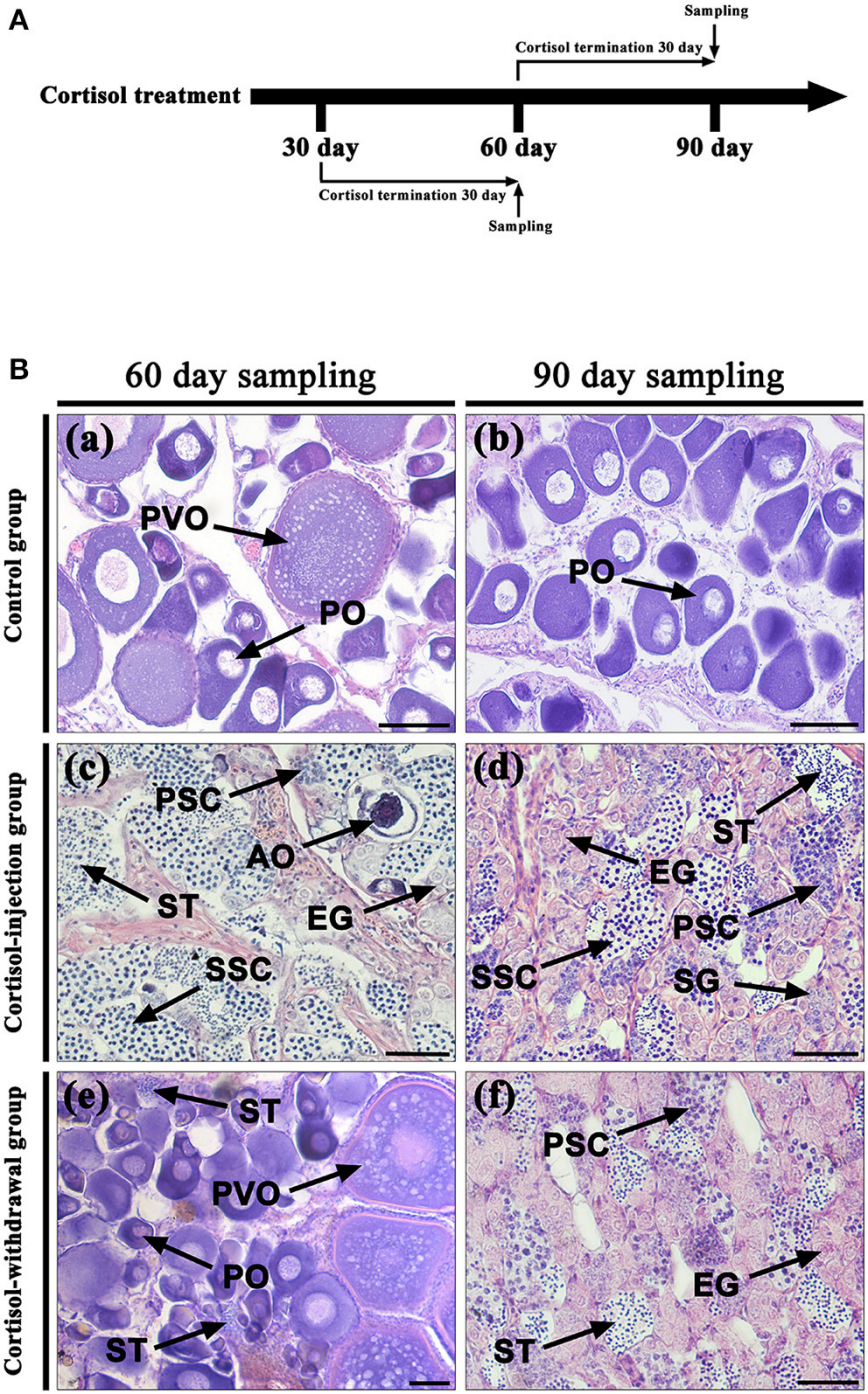
Gonad histology after cortisol withdrawal. **(A)** The time schedule of sampling. **(B)** Gonadal histology. (a,b) Gonadal histology of the control group at the 60 days after treatment (dat) and 90 dat sampling points, respectively; (c,d) gonadal histology of the cortisol-injection group at the 60 dat and 90 dat sampling points, respectively; (e) gonadal histology of the 30 days cortisol-withdrawal group at the 60 dat sampling point; (f) gonadal histology of the 60 days cortisol-withdrawal group at the 90 dat sampling point. AO, atretic oocyte; EG, early germ cell; PO, primary oocyte; PSC, primary spermatocytes; PVO, previtellogenic oocyte; SG, spermatogonia; SSC, second spermatocytes; ST, spermatid. Scale bars, 50 μm.

Figure 8A shows the time schedule of sampling. Table 3 shows the changes in gonadal stage across different groups of fish following the withdrawal of cortisol. In the 30 days cortisol-withdrawal experiment, the gonads of fish in the control group remained in the female phase. In contrast, the gonads of all fish in the cortisol-injection group and in the 30 days cortisol-withdrawal group had reached the intersex-transitional phase prior to cortisol withdrawal (Table 3). At the 60 dat sampling point, the gonads of fish in the control group contained numerous primary oocytes and previtellogenic oocytes (Figure 8B, a). In the cortisol-injection group, the gonad of one fish had almost reached the male phase, containing spermatogenic germ cells at various developmental stages and a few atretic oocytes (Figure 8B, c). The remaining three fish were in the intersex-transitional phase. However, in the cortisol-withdrawal group, only one fish remained in the intersex-transitional phase. In the other three fish, the gonads had been reversed to an ovarian status and featured primary oocytes and previtellogenic oocytes; the male germ cells had disappeared (Figure 8B, e).

**Table 3:** Gonadal stage of fish after the withdrawal of cortisol treatment.

**Groups**	***N***	**Time points of sampling**															
		**Day 0**				**Day 30**				**Day 60**				**Day 90**			
		**Gonadal stage**															
		**F**	**D**	**I**	**M**	**F**	**D**	**I**	**M**	**F**	**D**	**I**	**M**	**F**	**D**	**I**	**M**
Control	4	4				4				4				4			
Cortisol treatment	4	4						4				3	1			2	2
30-days cortisol withdrawal	4	4						4		3		1		3		1	
60-days cortisol withdrawal	4	4						4				1	3			3	1

*F, female phase; D, degenerative phase; I, intersex-transitional phase; M, male phase*.

In contrast, in the 60 days cortisol-withdrawal experiment, three of the four fish had reached the male phase prior to the withdrawal of cortisol (Table 3). At the 90 dat sampling point, the gonads of fish in the control group were characterized by the presence of primary oocytes (Figure 8B, b). Two of the four fish in the cortisol-injection group had completely changed sex to male (Figure 8B, d). In the cortisol-withdrawal group, one of the three sex-changed males had maintained male characteristics after 30 days of cortisol withdrawal (Figure 8B, f), while the other two had reverted to the intersex-transitional phase.

### Serum Steroid Hormone Levels After the Withdrawal of Cortisol

**Figure 9:**
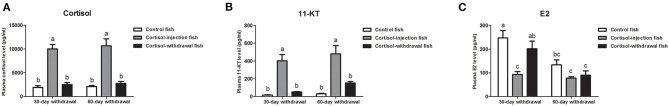
Serum steroid hormone levels after cortisol withdrawal. Changes in serum **(A)** cortisol levels, **(B)** 11-KT levels, and **(C)** E2 levels in the control fish, cortisol-injection fish, and cortisol-withdrawal fish at 30 days after cortisol withdrawal. Data from four fish samples are expressed as the mean ± SEM. Different letters above the bars indicate statistically significant differences (*P* < 0.05).

The levels of serum steroid hormones were further examined after the withdrawal of cortisol treatment. Compared with levels in the negative control fish, the fish receiving cortisol injections had significantly higher levels of serum cortisol and 11-KT (Figures 9A,B) and lower levels of serum E2 (Figure 9C) during the experiments. In the 30 days cortisol-withdrawal experiment, the fish receiving cortisol injection showed high levels of serum cortisol and 11-KT; in contrast, there was a significant decrease in serum cortisol and 11-KT levels in fish after 30 days of cortisol withdrawal. Levels in the cortisol-withdrawal fish were not significantly different from those in the negative control fish (Figures 9A,B). In contrast, serum E2 levels in the cortisol-withdrawal fish were significantly higher than those in the cortisol-injection fish but were not significantly different from those in the negative control fish (Figure 9C). In the 60 days cortisol-withdrawal experiment, we observed a significant decrease in serum cortisol and 11-KT levels in the cortisol-withdrawal fish (Figures 9A,B), but there was no significant change in serum E2 levels across the different treatments (Figure 9C).

### Gene Expression Profiles in the Gonad After Cortisol Withdrawal

**Figure 10:**
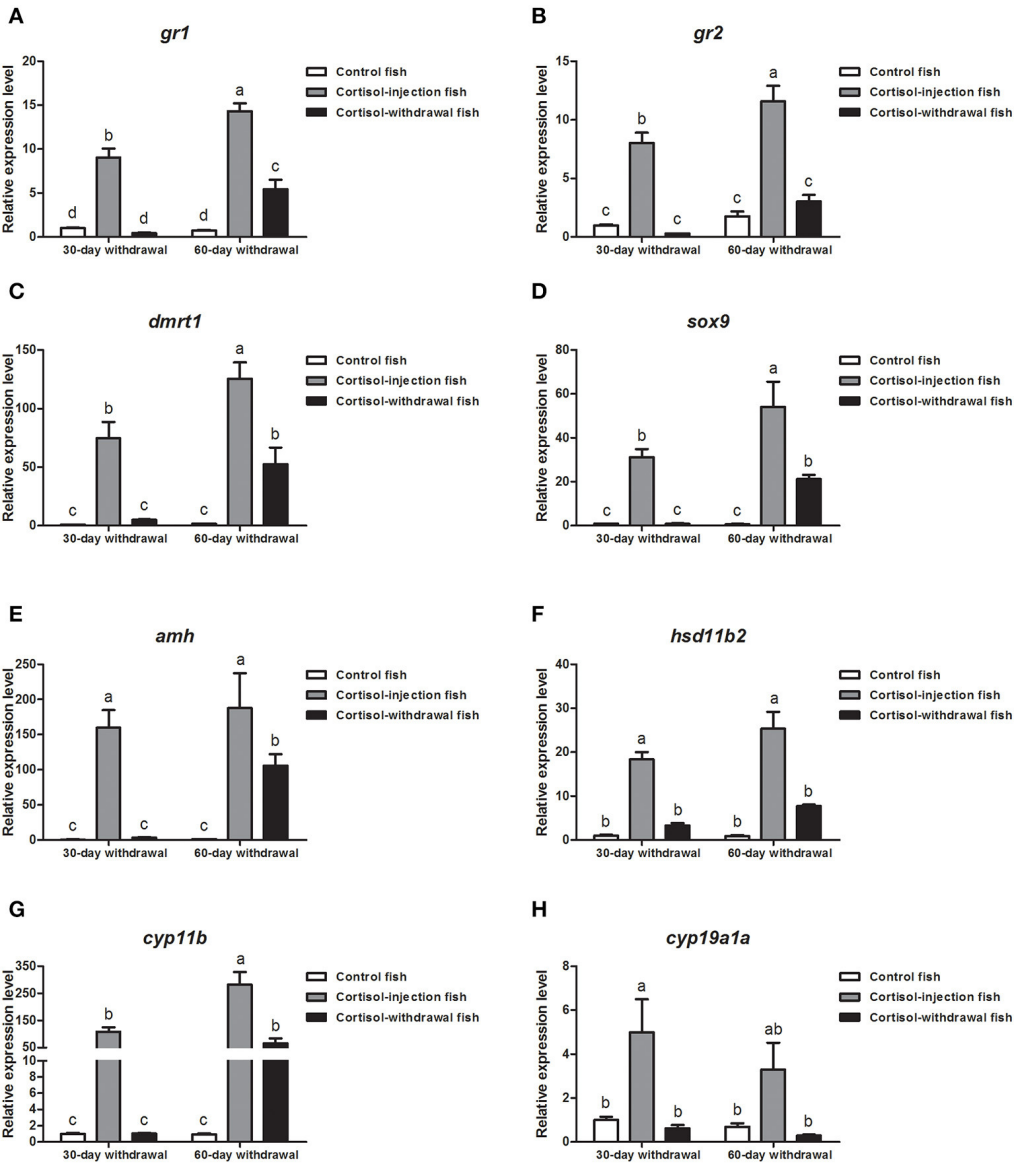
Expression profiles of key genes related to sex change in the gonad after cortisol withdrawal. **(A,B)** Expression profiles of glucocorticoid receptors *gr1* and *gr2*, respectively. **(C–G)** Expression profiles of male-related genes *dmrt1, sox9, amh, hsd11b2*, and *cyp11b*, respectively. **(H)** Expression profiles of female-related gene *cyp11a1a*. β*-actin* was used as the internal control. Data from four fish samples are expressed as the mean ± SEM for three replicates obtained from the samples. Different letters above the bars indicate statistically significant differences (*P* < 0.05).

Next, we investigated gene expression profiles after cortisol-injection withdrawal. In the 30 days cortisol-withdrawal experiment, the expression levels of the glucocorticoid receptor genes (*gr1* and *gr2*) and sex-related genes (*dmrt1, sox9, amh, hsd11b2, cyp11b*, and *cyp19a1a*) were significantly reduced in the cortisol-withdrawal fish and were not significantly different from those in the control fish after cortisol withdrawal (Figures 10A–H). Furthermore, in the 60 days cortisol-withdrawal experiment, the expression levels of *gr2, hsd11b2*, and *cyp19a1a* significantly decreased following the withdrawal of cortisol (Figures 10B,F,H). In contrast, although the levels of expression of *gr1, dmrt1, sox9, amh*, and *cyp11b* were significantly down-regulated after the withdrawal of cortisol, these levels were still significantly higher than those in the control fish (Figures 10A,C,D,E,G).

### Functional Analysis of Cortisol *in vitro*

**Figure 11:**
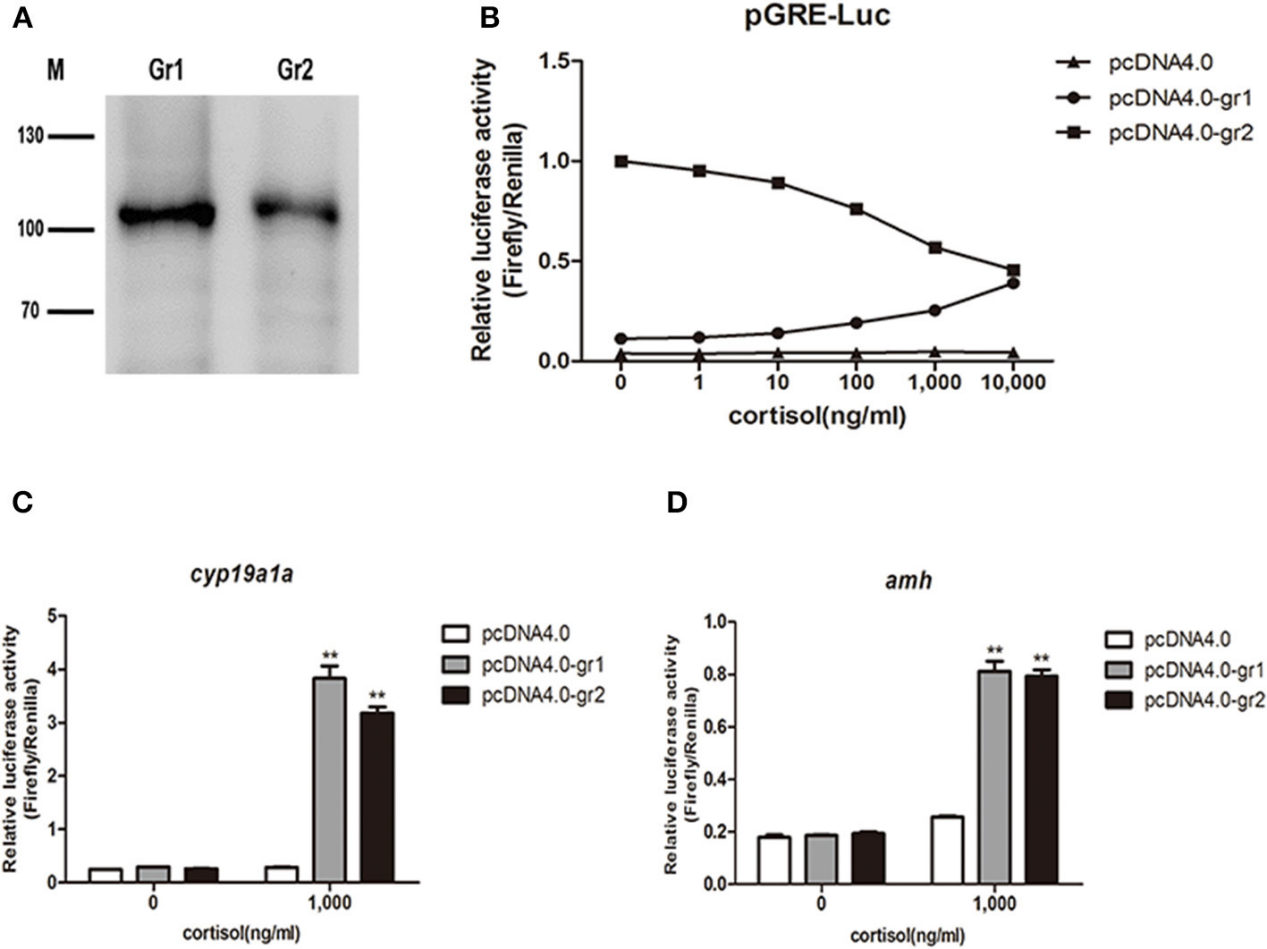
Luciferase activity in response to cortisol-stimulation *in vitro* receptor binding assay. **(A)** Overexpression of orange-spotted grouper GRs (GR1 and GR2) in HEK293 cells. Western blotting experiments were performed, and anti-his tag antibody was used. M indicates molecular marker in kDa. **(B)** Different cortisol concentrations induced GRE-driven luciferase activity in HEK293 cells. The construct overexpressing orange-spotted grouper GR1 or GR2 was transfected into HEK293 cells with a luciferase reporter gene (GRE-Luc). An empty construct without GR overexpression was used as the control. The cells were harvested for luciferase activity analysis after 12 h hormone treatment. **(C)** Effect of cortisol on the activity of the *cyp19a1a* promoter. The construct overexpressing GR1 or GR2 was transfected into HEK293 cells with *cyp19a1a* promoter construct. An empty construct without GR overexpression was transfected into HEK293 cells, with *cyp19a1a* promoter construct as the control. **(D)** Effect of cortisol on the activity of the *amh* promoter. The construct overexpressing GR1 or GR2 was transfected into HEK293 cells with *amh* promoter construct. An empty construct without GR overexpression was transfected into HEK293 cells, with *amh* promoter construct as the control. The cells were harvested for luciferase activity analysis after 12 h cortisol (1,000 ng/ml) treatment. Data are presented as the mean of four replicate wells ± SEM. The experiments were independently repeated three times. The asterisk ** indicates statistically significant difference (*P* < 0.05) compared with the control.

After confirming the expression of *gr1* and *gr2* in HEK293 cells (Figure 11A), the *in vitro* bioactivity of cortisol was assessed using a homologous receptor binding assay in HEK 293 cells transiently expressing orange-spotted grouper GRs. Using a GRE-driven luciferase assay, we found that luciferase activity exhibited a dose-response curve (Figure 11B). GR1 was activated by high concentrations of cortisol, while, in cells transfected with GR2, high concentrations of cortisol suppressed the expression of downstream GRE-driven luciferase (Figure 11B). We next examined whether the transcription of the female-related gene *cyp19a1a* and the male-related gene *amh* was regulated by cortisol/GR signaling in HEK 293 cells. Our data showed that a high concentration of cortisol (1,000 ng/ml) significantly stimulated the transcription of *cyp19a1a* and *amh* genes in cells transfected with GR1 or GR2 (Figures 11C,D).

## Discussion

In the present study, we demonstrated that a high dose of cortisol (50 mg/kg body weight) induced a change in sex (from female to male) in the protogynous orange-spotted grouper. All cortisol-treated groups exhibited degeneration of oocytes, although complete gonadal sex change was only observed in the high-dose cortisol group. However, none of the fish in the low-dose and medium-dose groups changed sex during the experimental period. In contrast, all fish in the high-dose group changed sex from female to male or remained in an intersex-transitional phase in which spermatogonial cells were accumulated but did not differentiate further. These results suggested that the effect of cortisol treatment on sex change was dose-dependent. Moreover, after the withdrawal of cortisol treatment, spermatogenesis in fish exhibiting the intersex-transitional phase and in some male fish immediately stopped, and developing sperm began to disappear; the gonads of some fish ultimately changed back to ovaries containing numerous primary oocytes and previtellogenic oocytes. In this study, only a few fish that had changed sex from female to male could be maintained after the withdrawal of cortisol. These results suggested that cortisol-induced sex change was not permanent.

In protogynous species, the degeneration of oocytes accompanied by a sharp drop in serum E2 levels and a simultaneous proliferation of spermatogonia accompanied by an increase of 11-KT levels are regarded as key events during female-to-male sex change (12, 20, 21). In the present study, mature female orange-spotted groupers were intraperitoneally injected with cortisol. Twenty-four hours after treatment, the mean levels of cortisol and 11-KT in the high-dose cortisol group were more than 10-fold greater than those in the control group, while E2 levels in the cortisol-treated fish did not change significantly. This result suggested that the elevation of cortisol levels could significantly enhance the synthesis of 11-KT. Previous studies in protogynous fish, such as ricefield eel (*Monopterus albus*), wrasse (*Thalassoma duperrey*), and honeycomb grouper, indicated that aromatase activity is important for maintaining the ovary and that a drop in E2 levels is necessary for the onset of female-to-male sex change (12, 13, 22). Interestingly, our present study showed that the expression of *cyp19a1a* was not downregulated by the administration of cortisol and showed no significant change during the process of cortisol-induced sex change. The reduction in E2 levels was only observed at 60 dat. Furthermore, an *in vitro* assay further demonstrated that cortisol stimulated but did not suppress transcription of the *cyp19a1a* gene. Collectively, these results suggest that the reduction in E2 levels is a result of ongoing masculinization rather than a trigger factor in the orange-spotted grouper. Moreover, a range of studies involving protogynous species have shown that the administration of 11-KT could induce complete sex change from female to male (23–25). Another study investigated the process of natural sex change in the protogynous honeycomb grouper (*Epinephelus merra*) and found that while serum 11-KT levels increased gradually, there was no significant reduction in E2 levels (22). These earlier reports supported the results of the present study in that cortisol might trigger sex change by increasing the production of 11-KT. Our results indicated that 11-KT plays a critical role in triggering protogynous sex change and that the elevation of 11-KT levels may be a better marker of the onset of sex change in the orange-spotted grouper than is a decline in E2.

Most teleost fish possess two glucocorticoid receptors, GR1 and GR2 (26). These receptors show significant differences in terms of hormone sensitivity and tissue expression in many species (27, 28). For example, in the rainbow trout (*Oncorhynchus mykiss*), GR1 is more sensitive to high levels of cortisol, while GR2 is more sensitive to lower levels (29). Cortisol acts upon different tissues by binding to intracellular receptors and by translocation to the nucleus, where it binds to GRE in the gene's promoter region and thus regulates transcription (3). This is known as the classical pathway by which cortisol modulates the transcription of target genes in fish (30). The results of the present study showed that both GR1 and GR2 were sensitive to cortisol. Interestingly, in the presence of cortisol, GR1 was stimulated, while GR2 suppressed the downstream GRE-driven expression of luciferase in a dose-dependent manner. However, our results also showed that the activation of GR2 stimulated the activity of the *cyp19a1a* and *amh* promoters, implying that GR2 might regulate downstream gene expression by the classical pathway in combination with an as yet, unidentified additional mechanism. In addition, the *in vitro* assays in this study were conducted in mammalian HEK 293 cells. Therefore, further studies are required to determine the mechanisms through which cortisol modulates the transcription of target genes in fish.

Cortisol-induced sex change is a continual process involving the degeneration of ovarian tissue and the reconstruction of testicular tissue during which the expression of male pathway genes (*dmrt1, sox9, amh, hsd11b2*, and *cyp11b*) increase significantly. *Cyp11b* and *hsd11b* are critical for the synthesis of 11-KT, the most potent androgen in teleosts (3). In the present study, the expression of *hsd11b2* was immediately up-regulated in all cortisol-treated groups after the administration of cortisol. Furthermore, in the high-dose cortisol group, the elevation of *hsd11b2* expression was observed throughout the process of cortisol-induced sex change; this coincided with an increase in serum 11-KT levels and the sex change process. In contrast, the upregulation of *cyp11b* was observed much later, and *cyp11b* expression was only evident in the high-dose cortisol group when the change of sex had been completed. Moreover, the downregulation of *hsd11b2* was accompanied by a reduction in serum 11-KT levels following the withdrawal of cortisol. These findings indicated that cross-talk between cortisol and 11-KT via the Hsd11b2 enzyme was crucial in protogynous sex change. Furthermore, following cortisol treatment, the expression of male-related genes increased significantly. Our *in vitro* assay also showed that cortisol could activate the transcription of the male-related gene *amh*. Previous research has shown that the overexpression of *amh* induces a female-to-male transition in the orange-spotted grouper (31).

We found that cortisol-induced sex change was not permanent in the orange-spotted grouper and could be reversed after the withdrawal of cortisol treatment. We found that cortisol activated the expression of sex-related genes, while the withdrawal of cortisol caused a marked reduction in the expression of these genes. Compared to the rapid reduction in gene expression in the 30 days withdrawal group, we found that the expressions of the male-related genes *dmrt1, sox9, amh*, and *cyp11b* in the 60 days withdrawal groups still remained higher than those in the control group. Following the withdrawal of cortisol, some sex-changed fish in the 60 days withdrawal group maintained a male sex identity, while all of the sex-changed fish underwent sex-reversal to a female identity in the 30 days withdrawal group. These results suggest that the sustained elevation of the expression of male pathway genes was closely associated with masculinization in the orange-spotted grouper. Consequently, a prolonged treatment period may be necessary for inducing permanent sex change in response to cortisol treatment.

In conclusion, our study demonstrated that the long-term administration of cortisol induces gonadal sex change in the protogynous orange-spotted grouper. Our results strongly suggest that cortisol triggers masculinization by increasing the production of 11KT and directly activating the expression of sex-related genes. Further studies are now required to identify the role of cortisol in the natural process of sex change induced by environmental factors, and to determine the exact mechanisms underlying the transduction of environmental signals into the molecular cascade and thus triggering a change in sex.

## Data Availability Statement

The datasets for this study can be found in the GEO repositories, The accession number is GSE142814.

## Ethics Statement

The animal study was reviewed and approved by Animal Research and Ethics Committees of Sun Yat-sen University.

## Author Contributions

JC, LX, SL, HL, and YZ designed the experiments. JC, CP, ZY, and QY conducted all the experiments and analyzed the data. LX, SL, HL, and YZ designed the experiments. HZ provided the required facilities and support services. JC wrote the manuscript. All authors read the final article and approved its submission.

### Conflict of Interest

The authors declare that the research was conducted in the absence of any commercial or financial relationships that could be construed as a potential conflict of interest.
